# Orthokeratinized Odontogenic Cyst: A Report of Three Clinical Cases

**DOI:** 10.1155/2013/672383

**Published:** 2013-09-26

**Authors:** María del Carmen González Galván, Abel García-García, Eduardo Anitua-Aldecoa, Rafael Martinez-Conde Llamosas, José Manuel Aguirre-Urizar

**Affiliations:** ^1^Oral and Maxillofacial Pathology Unit, Dental Clinic Service, Stomatology II Department, UFI 11/25, University of the Basque Country (EHU/EHU), Vizcaya, 48940 Leioa, Spain; ^2^University of Santiago of Compostela, 15782 Santiago of Compostela, Spain; ^3^Private Practice, Eduardo Anitua Institute, 01005 Vitoria-Gasteiz, Spain

## Abstract

The orthokeratinized odontogenic cyst (OOC) is a rare developmental odontogenic cyst that has been considered as a variant of the keratocystic odontogenic tumour (KCOT) until Wright (1981) defined it as a different entity. Surgery is the usual treatment, and recurrence or association with Gorlin-Goltz syndrome has rarely been described. In this report, we presented three cases of this pathology, and we review the principal clinical, histological, radiological, and therapeutic aspects. *Case 1*. A 73-year-old female presents with a slight swelling on the right mandible, associated with an unilocular well-defined radiolucent lesion. *Case 2*. A 27-year-old female presents with a painful mandibular swelling associated with an unilocular radiolucent lesion posterior to the 4.8. *Case 3*. A 61-year-old male was casually detected presents with an unilocular radiolucent lesion distal to the 4.8. *Conclusion*. The OOC is a specific odontogenic clinicopathological entity that should be differentiated from the KCOT as it presents a completely different biological behaviour.

## 1. Introduction

The orthokeratinized odontogenic cyst (OOC) is a developmental odontogenic cyst relatively rare, arising from the cell rests of the dental lamina [1, 2]. It was first described by Schultz in 1927 [3] as an orthokeratinized variant of the formerly called odontogenic keratocyst, today known as the keratocystic odontogenic tumour. It is not until 1981 that Wright defines this as an independent entity [4].

Since then it has received various designations, such as “orthokeratinized variant of odontogenic keratocyst” or “orthokeratinized cyst of the mandible”. It is not until 1998 that Li et al. suggest the term “orthokeratinized odontogenic cyst,” which is the most accepted at the present time [2, 5].

The OOC occurs predominantly in males between the third and fourth decades, with a mean age of 33.5 years [2, 5]. The lesion is located mainly in the molar region and posterior mandible [1, 2]. These tumours can reach a large size that causes cortical expansion and presents as a swelling, along with pain, although in most cases it can be detected incidentally during a radiographic examination [1, 2].

Radiographically the cyst appears as a well-circumscribed, unilocular, or multilocular radiolucency that occasionally is associated with an unerupted tooth or with the root without causing resorption [1, 6, 7]. Displacement of neighbouring teeth and of the inferior dental canal has been described [5].

The histopathological analysis confirms the diagnosis which shows a cystic cavity lining composed of a thin and uniform stratified squamous epithelium with a thick granular layer and orthokeratin [1, 8, 9]. 

A differential diagnostic feature with the keratocystic odontogenic tumour is that no cases of OOC have been associated with nevoid basal cell carcinoma syndrome [8, 10].

Surgical enucleation is the treatment of choice for the OOC, and low recurrence rate has been described between 0 and 2% of cases which is in marked contrast with the 40% recurrence of KCOT [2, 11, 12].

On this paper, we report three clinical cases of the orthokeratinized odontogenic cyst and a review on the main clinicopathological aspects.

## 2. Clinical Cases

**Case 1**

**Figure 1:**
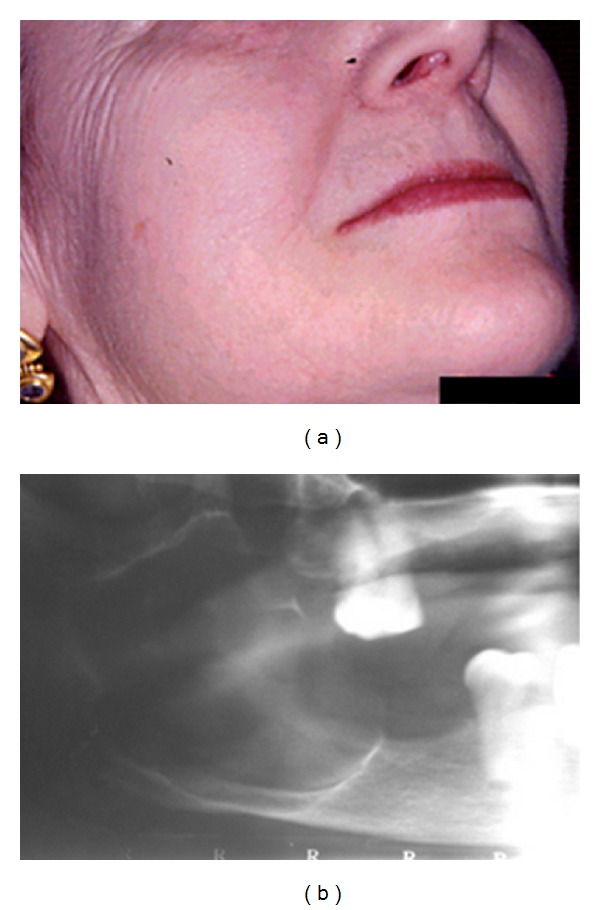
(a) Clinical image. (b) Multilocular radiolucent lesion.

A 73-year-old female was referred presenting a slight asymptomatic swelling on the right mandibular angle and ascending ramus of years of evolution (Figure 1(a)). The patient was under treatment with Diclofenac and Piroxicam for arthrosis symptoms and reported no toxic habits.

Radiographically, the lesion appeared as a well-defined multilocular radiolucency on the right posterior mandible and ascending ramus (Figure 1(b)). The computed tomography showed an expansive multilocular and well-defined radiolucency. A fine needle aspiration cytology (FNAC) was performed, and a pearly-white creamy liquid material was obtained. The cytology of the material confirmed the presence of squamous cells.

The complete enucleation of the lesion was performed under general anesthesia, and the material obtained was analyzed for its histopathological figures. Histopathological examination of excised tissue revealed the presence of cystic cavity integrated by dense connective tissue with an inner lining of thin and uniform epithelium with marked granular layer and hyperorthokeratosis. Based on the clinical, radiological, and histological data, a diagnosis of orthokeratinized odontogenic cyst was made. After eleven years of follow-up, no sign of recurrence has been noted.

**Case 2**

**Figure 2:**
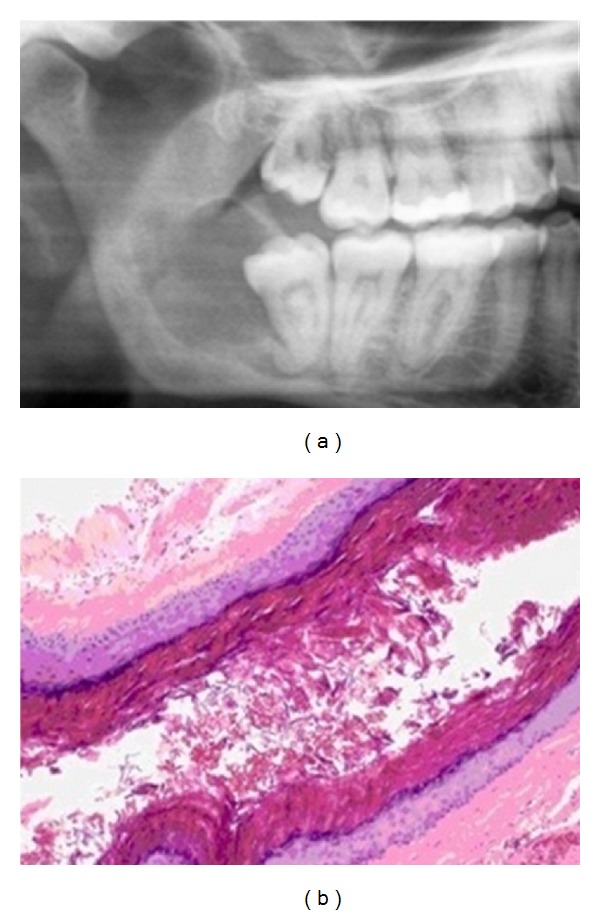
(a) Unilocular radiolucent lesion distal to 4.8. (b) Fibrous connective tissue with a stratified epithelium with orthokeratosis lining.

A 27-year-old female presents with a slightly painful swelling in the region of the right inferior third molar of 15 days of evolution, with no other clinical data of interest, nor does she report toxic habits. The orthopantomography shows an unilocular radiolucent lesion, posterior to the 4.8, extending to the ascending ramus (Figure 2(a)). The lesion produced displacement of the molar without causing root resorption. The computed tomography showed the lesion and a slight expansion of the vestibular and lingual walls.

The extraction of the 4.8 and nucleation of the lesion were made under general anaesthesia. In the histopathological analysis, a cystic lesion was recognized with a fibrous connective tissue capsule without inflammation, covered by a stratified epithelium with orthokeratosis and marked granular layer (Figure 2(b)). With the data, the diagnosis of orthokeratinized odontogenic cyst was given.

After the excision of the lesion and the third molar extraction, the pain disappeared.

The outcome was favourable, and after 15 months, no signs of recurrence were recognized.

**Case 3**

**Figure 3:**
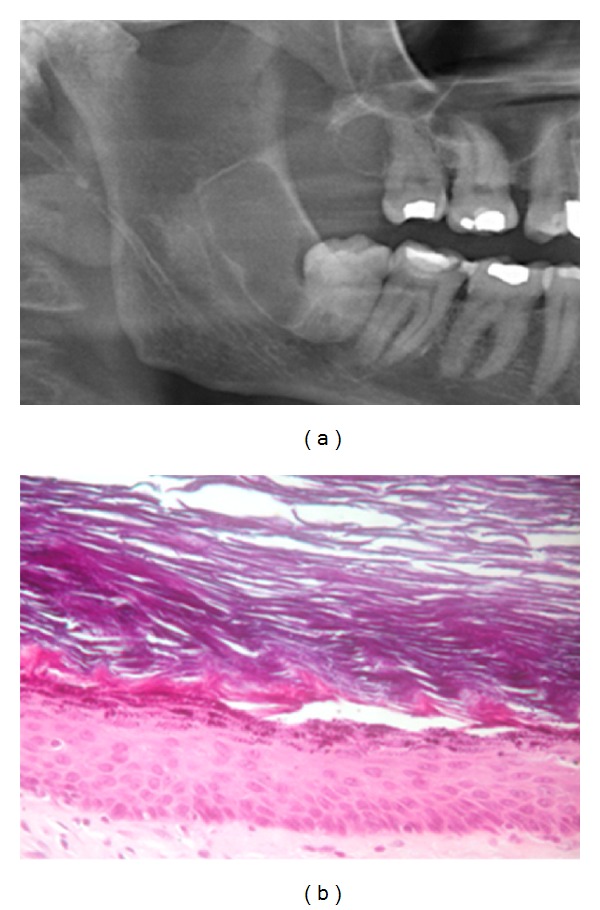
(a) Initial orthopantomography. (b) Fibrous connective tissue with a stratified epithelium with hyperorthokeratosis lining.

A 61-year-old male who was on a routine dental follow-up presents with a well-defined radiolucent lesion, posterior to the right inferior third molar and extended to the ramus (4.8) on the radiographic exploration (Figure 3(a)). The patient did not show any other signs of interest neither toxic habits. The extraction of the 4.8 and enucleation of the lesion were performed under local anaesthesia. On the histopathological analysis, a cystic lesion was recognized with a fibrous connective tissue capsule with no inflammation, covered with a lining of a thin, uniform hyperkeratotic stratified epithelium with marked granular layer.

The cavity was filled with keratin flakes (Figure 3(b)). With this data, the diagnosis of orthokeratinized odontogenic cyst was established.

The outcome was favourable, and after 9 months, no signs of recurrence have been recognized.

## 3. Discussion

The orthokeratinized odontogenic cyst is an infrequent developmental cyst that occurs most often between the fourth and fifth decades and with a male gender predilection (3, 2 : 1) [1, 2]. Curiously in our cases, two were women and one of them with an advanced age, which we believe to be slow-growing and chronic lesion in an area where extractions were performed without a correct curettage and probably with no previous radiography [1, 5, 11].

As in most cases described previously, our lesions were located in the posterior mandible and extend to the ascending ramus [2, 5, 11]. Swelling is the most frequent symptom and is accompanied on occasions with pain, although in most cases described, the lesion was asymptomatic. The presence of paresthesia, as in Case 2, is a rare sign and is associated with irritative phenomena, such as the infection associated with the impacted third molar [1, 2, 11]. 

Radiographically, the OOC may appear as a well-defined, unilocular or multilocular, radiolucent solitary lesion associated frequently with impacted teeth, and it on occasions can displace the inferior alveolar nerve.  Case 1 presented as a large lesion not associated with teeth. We believe it was slow growing of long evolution. In Cases 2 and 3, the lesions were more typical and related to an impacted molar, located distal to the 4.8 and with no signs of resorption or displacement [2, 7, 11].

Histologically, the OOC shows a cystic cavity lined by a regular stratified squamous epithelium, usually thin and uniform about 4- to 9-cell layers thick. This epithelium presents a defined basal layer that exhibits palisade cuboidal or flat cells, with nuclear hyperchromatism, an intermediate layer of polyhedral cells with eosinophilic cytoplasm, and a thick superficial layer of orthokeratin [1, 2, 8]. This entity must be differentiated from the KCOT that shows a regular epithelium of 5- to 10-cell layers thick with the basal cells lined with an elongated nucleus and the presence of a characteristic superficial corrugated layer of parakeratin [13, 14].

In relation to the immunohistochemical pattern, the OOC does not show activity of the epithelial membrane antigen (EMA) and of the carcinoembrionary antigen (CEA). Moreover, the level of expression of Ki-67 and p53 is lower than on KCOT that suggests a reduced proliferative activity [1, 2, 10]. The reactivity to cytokeratins has showed differences, as OOC stains to cytokeratins 1, 2, and 10 which would suggest a normal differentiation of the epidermis whilst the KCOT reacts to cytokeratins 4, 13, 17, and 19, demonstrating that these are different entities [15].

The differential diagnosis of the OOC includes other radiolucent lesions of the jaws, mainly odontogenic lesions such as dentigerous cyst or paradental cyst [1, 5]. Odontogenic tumours such as ameloblastoma and KCOT should be included. The OOC presents similar radiographic characteristics with the ameloblastoma and the KCOT, such as its tendency to involve the mandibular angle or to appear as a multilocular radiolucency. Unlike these entities, the OOC does not cause root resorption, which is a frequent characteristic on ameloblastomas and KCOT [5].

Conservative surgical removal with complete enucleation of the cystic lesion seems to be the treatment of choice [1, 5, 7].

 All our cases were removed with conservative enucleation and have had a periodical follow-up with no recurrence reported to date. 

We conclude that the orthokeratinized odontogenic cyst is an independent clinical and pathological entity of the keratocystic odontogenic tumour (KCOT) with a different prognosis. This lesion should be included in differential diagnosis of the radiolucent lesions of the jaws, preferably of the mandibular lesions associated with an impacted tooth.
